# Going beyond psychopathology—positive emotions and psychological resilience

**DOI:** 10.4103/0019-5545.58887

**Published:** 2010

**Authors:** G. Swaminath, B. R. Ravi Shankar Rao

**Affiliations:** Department of Psychiatry, M S Ramaiah Medical College, Bangalore - 560 054, India

‘It was quite sudden. I was sitting alone in a room on the first floor of my uncle's house. I seldom had any sickness, and on that day there was nothing wrong with my health, but a sudden violent fear of death overtook me. There was nothing in my state of health to account for it, and I did not try to account for it or to find out whether there was any reason for the fear. I just felt “I am going to die” and began thinking what to do about it. It did not occur to me to consult a doctor or my elders or friends; I felt that I had to solve the problem myself, there and then.

The shock of the fear of death drove my mind inwards and I said to myself mentally, without actually framing the words: “'Now death has come; what does it mean? What is it that is dying? This body dies.” And I at once dramatized the occurrence of death. I lay with my limbs stretched out stiff as though rigor mortis had set in and imitated a corpse so as to give greater reality to the enquiry: I held my breath and kept my lips tightly closed so that no sound could escape, so that neither the word “I” nor any other word could be uttered. “Well then, “I said to myself, “this body is dead. It will be carried stiff to the burning ground and there burnt and reduced to ashes. But with the death of this body am I dead? Is the body ‘I’? It is silent and inert but I feel the full force of my personality and even the voice of the ‘I’ within me, apart from it. So I am Spirit transcending the body. The body dies but the Spirit that transcends it cannot be touched by death. That means I am the deathless Spirit.”[1]

## TRUE GRIT

The yogic path of self-enquiry and the enlightenment process of the 17-year-old Ramana (later Ramana Maharishi of Tiruvannamalai), took place in a flash and he emerged a *jnana yogi*, of the highest order, with a large following, many of whom visit his ashram for meditation and peace. Carl Jung spoke highly of Ramana's realisation, and also of its being typically Indian, with its emphasis on the identification of the Self with God.[1]

Such an experience would be customary for many of our anxious patients. The intense, sudden violent fear of death, along with anticipatory anxiety of recurrence and avoidance, drives many to seek emergency solace at hospitals, as well as long-term management using prescription drugs which reduces these symptoms. The extraordinary psychological resilience of Ramana in being able to overcome this intense fear through self-observation, sublimation and asceticism to emerge a superior being is legendary as well as noteworthy. Affective style, one of the most salient characteristics of emotion, is the extraordinary heterogeneity in how different individuals respond to the same emotionally provocative challenge.[2]

The focus of mental healthcare has too often been on understanding and alleviating negative emotional states, such as anxiety, depression, abuse, prejudice and disharmony among others. Focus was lacking on how a majority of people manage to live lives of dignity and purpose, despite all difficulties, and this is now being addressed.[3]

‘Positive psychology’ is the study of ordinary human strengths and virtues.[3] It asks, “What is the nature of the effectively functioning human being, who successfully applies evolved adaptations and learned skills?”.[3] Positive psychology recognizes that persons who carry even the weightiest psychological burdens, can care about much more in their lives, than just the relief of their suffering.[4] Troubled persons often want more satisfaction, contentment, and joy, not just less sadness and worry. They want to build their strengths, not just correct their weaknesses, or removal of their suffering.[4] They want lives imbued with meaning and purpose.[4] The fostering of positive emotion and the building of character may help—both directly and indirectly—to alleviate suffering and to undo its root causes.[4]

Beyond the extraordinary physical and financial devastation and loss of human life, the September 11 attacks generated considerable emotional turmoil among US citizens. Early polling revealed crying spells,[5,6] anger,[5,6] sadness, fear, anxiety,[5,6] decreased sleep,[7] difficulty in concentration,[7] and a loss of personal sense of safety and security[5] in about 50 to 70% of those polled.[5] Amidst this amalgam of negative emotions, positive emotions seem unwarranted, even inappropriate. However, positive emotions are known to co-occur alongside negative emotions during stressful circumstances.[8] Several polls indicated that US citizens reported showing more affection for family members and relatives, with 60% reporting that their personal relationships were strengthened following the attacks.[9]

## ‘BROADEN-AND-BUILD’

It is clear that positive emotions such as gratitude, interest, and love provide more pleasant subjective experiences than do negative emotions such as anger, sadness, fear, and anxiety.[6] To the extent that positive emotions reduce the focus on negative emotions, they can put people's minds at ease. In addition, positive emotions act as active ingredients in superior coping and thriving, despite adversity.[6]

‘Positive emotions prompt individuals to engage with their environments and partake in activities’, writes Fredrickson, which are adaptive to the individual and species, motivating them to approach and explore.[10] Positive emotions, in her words, “broaden and build” i.e. they broaden people's momentary thought-action repertoires and build their enduring resources, ranging from physical and intellectual resources to social and psychological ones.[10] Negative emotions produce specific action tendencies (narrowing of a person's momentary thought-action repertoire) e.g. to escape (from fear), attack (when angry) or expel (when disgusted) which promotes quick and decisive action assisting survival.[10] In contrast, positive emotions do not bring immediate adaptive benefits, but build personal resources which can be drawn on to manage future threats.[10] For example, joy broadens by creating the urge to play, push the limits and be creative; interest broadens by creating the urge to explore, take in new information and experiences; contentment broadens by creating the urge to savor current life circumstances and integrate these circumstances into a new view of self and the world; pride broadens by creating the urge to share news of achievements and love (an amalgam of joy, interest and contentment) broadens by creating recurring cycles of urges to play with, explore, and savor experiences with loved ones thus improving on habitual modes of thinking and behaviour.[10]

## TANGIBLE BENEFITS

The benefits of positive emotions are: i) Physiological undoing, i.e. physiologically down-regulate lingering negative emotions[11] ii) Cognitive broadening i.e. cognitive broadening which expands and improves the ways people cope during crises[6] and iii) Resource building i.e. the broadening triggered by positive emotions builds a range of personal resources, including physical resources (e.g., physical skills, health, longevity), social resources (e.g., friendships, social support networks), intellectual resources (e.g., expert knowledge, intellectual complexity), and psychological resources (e.g., resilience, optimism, creativity).[6]

Recurrent experiences of positive emotions results in promotion of two healthy traits: psychological resilience, and positive emotional granularity. Psychological resilience is the flexibility in response to changing situational demands, and the ability to bounce back from negative emotional experiences.[11] Resilient individuals experience positive emotions even in the midst of stressful events, which may explain their ability to rebound successfully despite adversity. These individuals bounce back from negative emotional arousal physiologically as well.[11] Resilient people may understand the benefits associated with positive emotions and use this knowledge to their advantage when coping with negative emotional events.[11] Positive emotions appeared to aid resilient individuals in their ability to build psychological resources that are essential for coping effectively with large-scale tragedy. Thus the positive emotions experienced by resilient people may serve as protective factors useful in promoting short-term health benefits as well as long-term advantages for coping in the future.[11]

There are individual differences in how people verbally report their affective experiences, with highly granular individuals reporting their emotional experience in differentiated terms with discrete emotion labels (happy, content, sad, angry, etc.) to capture their distinctiveness, in contrast to low granular individuals who express emotional experiences in a more global and undifferentiated manner (feeling good or feeling bad).[11] Those individuals with higher positive emotional granularity i.e. who represent positive emotional experiences with precision and specificity, are less likely to mentally self-distract during stressful times, are more engaged in the coping process, are less automatic in their responding, and are more likely to think through their behavioral options before acting.[11] While coping is traditionally seen as “reactive” or something that occurs temporarily after a stressful event, the approaches to coping reported by individuals with positive emotional granularity seem “proactive” and future-oriented, in that the individual takes preparatory steps before acting on stress.[11] Thus, by prompting one to scan one's array of coping options, positive emotional granularity may afford an individual with the ability to stretch capacities for regulating negative emotional experiences. Positive emotional granularity, then, may be a mechanism by which resilient people achieve superior coping abilities.[11]

There has been recent research into the beneficial effects of positive emotions on patients suffering from burns,[12] heart disease,[11] cancer[11] and depression.[13] Positive emotions can be an important factor that buffers individuals against maladaptive health outcomes.[11] Enhancing positive emotions in psychotherapy, ‘Positive psychotherapy’ has also been recommended as a novel way to treat and prevent depression.[13] Positive emotions predict happiness, life satisfaction, improved coping resources and desirable life outcomes.[14] Amidst the emotional turmoil generated by the September 11 terrorist attacks, subtle and ‘fleeting experiences of gratitude, interest, love, and other positive emotions appeared to hold depressive symptoms at bay and fuel post crisis growth’.[6] Mental health professionals need to raise their competence beyond ameliorating the suffering from illness to focusing more attention on these positive aspects of human nature.
